# Bactericidal Anti-Adhesion Potential Integrated Polyoxazoline/Silver Nanoparticle Composite Multilayer Film with pH Responsiveness

**DOI:** 10.3390/polym14173685

**Published:** 2022-09-05

**Authors:** Xiaojiong Bao, Xiaofei Huang, Xiaoqiang Jin, Qiaoling Hu

**Affiliations:** 1 Department of Polymer Science and Engineering, Zhejiang University, Hangzhou 310027, China; 2 The Second Affiliated Hospital of Zhejiang University School of Medicine, Zhejiang University, Hangzhou 310009, China

**Keywords:** polyoxazoline, silver nanoparticles, multilayer film, self-assembly, pH responsiveness

## Abstract

Bacterial infections occur frequently during the implantation of medical devices, and functional coating is one of the effective means to prevent and remove biofilms. In this study, three different hydrophilic polyoxazolines with carboxyl groups (aPOx: PT1, PT2 and PT3) and bactericidal silver nanoparticles (AgNPs) were synthesized successfully, and an aPOx-AgNP multilayer film was prepared by electrostatic layer-by-layer self-assembly. The effect of charge density and assembly solution concentration was explored, and the optimal self-assembly parameters were established (PT2 1 mg/mL and AgNPs 3 mg/mL). The hydrophilicity of the surface can be enhanced to resist protein adhesion if the outermost layer is aPOx, and AgNPs can be loaded to kill bacteria, thereby realizing the bactericidal anti-adhesion potential integration of the aPOx-AgNP multilayer film. In addition, the aPOx-AgNP multilayer film was found to have the characteristic of intelligent and efficient pH-responsive silver release, which is expected to be used as a targeted anti-biofilm surface of implantable medical devices.

## 1. Introduction

The frequent occurrence of various metabolic diseases, such as cardiovascular and cerebrovascular diseases, has led to an increasing number of medical device implantation operations [1,2]. However, during the implantation of external devices into the body, bacteria easily adhere to the surface of the device and form films of bacterial aggregates, i.e., biofilms [3,4,5]. The formation of bacterial biofilms is a dynamic process that can be divided into four stages: the colonization stage of reversible bacterial adhesion, the aggregation stage of irreversible adhesion, the mature stage of biofilms, and the shedding and recolonization stage of bacteria [6,7,8]. Compared with free bacteria, bacteria in biofilms are significantly more resistant to antibiotics and bactericidal agents, which cannot be easily phagocytosed by macrophages, nor can they be eliminated by the body’s immune system. Therefore, preventing the formation of biofilms, especially at the early colonization stage of reversible bacterial adhesion, rather than killing and removing already-formed biofilms, helps to reduce the occurrence of bacterial infection more simply and efficiently, as well as control the failure rate of implantation surgery at the source.

Functional coating is one of the effective means to construct anti-biofilm surfaces, which are mainly divided into two categories according to the mechanism of action [9,10,11,12,13]: anti-adhesion surfaces and bactericidal surfaces. (1) Anti-adhesion surfaces against bacterial adhesion proteins can also be subdivided into hydrophilic surfaces, superhydrophobic surfaces and slip surfaces according to the mechanism of action [14]. Among them, the construction of hydrophilic surfaces is the most common path, whose mechanism of anti-adhesion is often attributed to the formation of a surface hydration layer, and hydrophilic molecules are often used for the construction of such surface hydration layers [15,16,17]. Polyoxazoline (POx), with a polypeptide-like structure, is hydrophilic and tends to form a hydration layer upon contact with tissue fluid, which has been a promising option for current functional surfaces [18,19,20,21,22]. It has the advantages of greater molecular chain flexibility, easier functionalization of end groups and adjustable hydrophilicity, so it is considered as a potential substitute for polyethylene glycol. (2) Bactericidal surfaces can kill bacteria directly through adequate release or effective contact of antibiotics or bactericidal agents. Silver nanoparticles (AgNPs) have been extensively studied and applied as broad-spectrum, high-efficiency, low-toxicity bactericidal agents [23,24,25,26]. AgNPs have excellent bactericidal properties against both Gram-positive bacteria (Staphylococcus aureus) and Gram-negative bacteria (Escherichia coli) [27,28,29].

Both anti-adhesion surfaces and bactericidal surfaces play important roles in different stages of biofilm formation, as mentioned above, but neither can resist the formation of biofilms completely. It is difficult for a single antibacterial method to effectively and persistently deal with the complex and changeable environment in the four stages of biofilm formation. Therefore, the integration of anti-adhesion surfaces and bactericidal surfaces has emerged to resist bacterial adhesion and kill adherent bacteria through a synergistic effect [30,31,32]. A multilayer film is an excellent choice to leverage the above synergistic effect, which can be prepared by a physical deposition method, chemical deposition method, lamination method, coating method or self-assembly method [33,34]. Among them, electrostatic layer-by-layer self-assembly is widely used to make connections between metal nanoparticles and polymers with opposite charges with a simple operation for bactericidal anti-adhesion integrated materials [35,36,37,38]. In addition, smart stimuli-responsive surfaces, with the help of changes in the microenvironment of the bacterial infection site, such as local acidification, can achieve a more targeted release of antibiotics or bactericidal agents and apparently reduce the dosage and toxicity of bactericidal agents [39,40,41].

**Figure 1:**
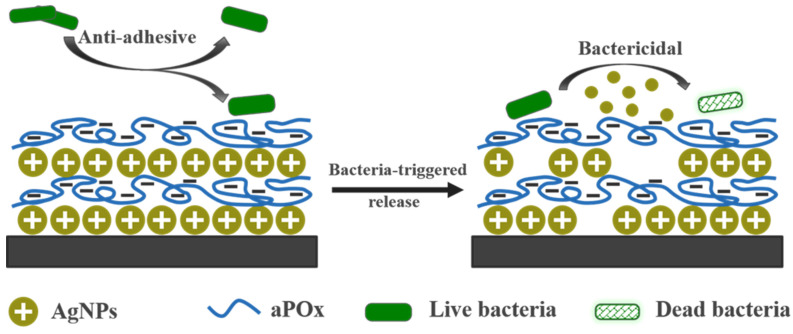
Scheme of the bactericidal anti-adhesion potential integrated aPOx-AgNP multilayer film.

In this study, a series of hydrophilic POx with carboxyl groups (aPOx) were synthesized after monomer synthesis, polymerization and post-polymer modification. Bactericidal AgNPs were prepared by reductive stabilization of polysaccharides. Negatively ionized aPOx can interact with positively charged moieties in AgNPs, and aPOx-AgNP multilayer film was prepared by electrostatic layer-by-layer self-assembly (Figure 1). In order to study the effect of the charge density of POx on the assembly, the copolymerization feed ratio of monomers was adjusted to prepare aPOx with different carboxyl group ratios, as well as different negative charge ratios, and the self-assembly behavior of the aPOx-AgNP multilayer film was tracked by QCM with the optimization of self-assembly parameters. The silver content, silver release, surface hydrophilicity, film thickness and other properties of aPOx-AgNP multilayer films were characterized by a UV spectrophotometer, in vitro silver release test, contact angle analyzer, SEM and ellipsometer.

## 2. Experiments

### 2.1. Materials

Chitosan (Mw 30 kDa, deacetylated degree 96%) was purchased from Zhejiang Golden Shell Pharmaceutical Co., Ltd.; 3,4-dihydroxy phenylpropionic acid, 1-ethyl-3-(3-dimethylaminopropyl)-carbodiimide hydrochloride and branched polyethyleneimine (BPEI, Mw 25 kDa) were purchased from Sigma-Aldrich. Silver nitrate (AgNO_3_) was purchased from Acros Organics. All other chemical reagents were purchased from Sinopharm Chemical Reagent Co., Ltd. and were of analytical grade. All aqueous solutions were prepared with distilled and deionized water (DDW).

### 2.2. Synthesis and Characterization of aPOx (PT1, PT2 and PT3)

**Figure 2:**
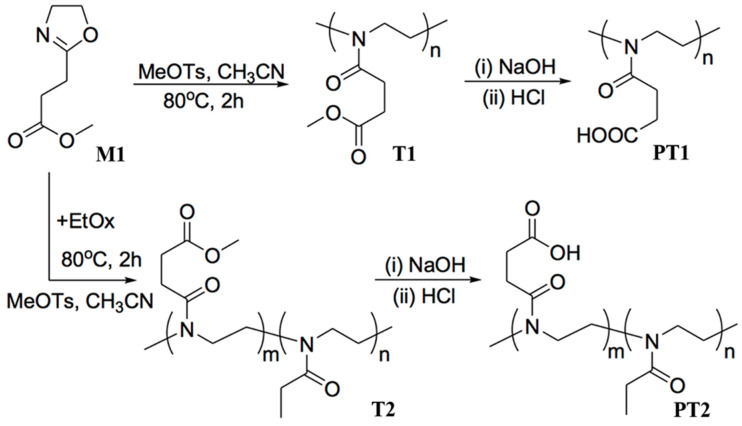
Molecular formulas of M1, P1, P2, PT1 and PT2.

The main chain of POx is electrically neutral, and as long as the side chain is negatively charged, it can electrostatically assemble with positively charged particles. In this study, the carboxyl group was selected as the negatively charged group, and 2-(2-methoxycarbonyl)ethyl-2-oxazoline (M1) was synthesized instead of 2-ethyl-2-oxazoline (EtOx) as the polymer monomers [42,43]. Through homopolymerization and post-modification of the monomers, the obtained aPOx side chains were all carboxyl groups. At the same time, in order to study the effect of the charge density of aPOx on the assembly, the copolymerization feeding ratio of two monomers, M1 and EtOx, was adjusted to obtain aPOx with different carboxyl group ratios as well as different negative charge ratios. Homopolymer P1 and copolymers P2 and P3 were modified to obtain PT1, PT2 and PT3, respectively. The molecular formula is shown in Figure 2.

**Figure 3:**
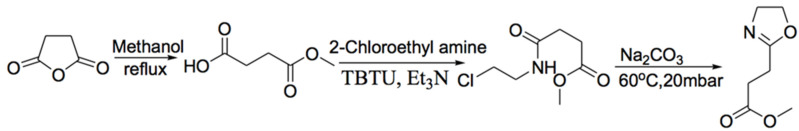
Synthetic route of M1.

(1) Monomer synthesis: The synthesis of M1 is divided into three steps: the succinic anhydride ring-opening reaction, amidation reaction and oxazoline ring-closing reaction. The synthetic route is shown in Figure 3.

Succinic anhydride ring-opening reaction: Succinic anhydride (50.0 g, 0.5 mol) and methanol (30.3 mL, 0.75 mol) were added to a 250 mL three-necked round-bottom flask with a spherical condenser. After the condensed water was turned on, the temperature was raised to 60 °C, and the solution was stirred for 3 h. After the heating was stopped, the liquid crude product was transferred to a beaker while it was still hot and stirred and cooled with a glass rod, and the crude product was smashed after solidification. Pure ethyl acetate was used to recrystallize it at 60 °C to obtain a colorless and transparent crystalline solid, which was the intermediate product, monomethyl succinate.

Amidation reaction: A 500 mL three-necked round-bottom flask was baked with nitrogen protection, and monomethyl succinate (13.2 g, 0.1 mol), 2-chloroethylamine hydrochloride (12.8 g, 0.11 mol) and tetramethylurea tetrafluoroborate (TBTU, 35.5 g, 0.11 mol) were added to the flask and dissolved in 150 mL of dichloromethane. After a sufficient ice bath, triethylamine (41.6 mL, 0.3 mol) was added dropwise to the solution, and it was stirred under nitrogen protection for 4 h. The reaction solution was then extracted 3 times with DDW, and the organic phase was retained and dried by adding 20.0 g of anhydrous magnesium sulfate for 1 h. The crude product was obtained as a yellow viscous liquid by filtration and spin-drying of the filtrate.

Oxazoline ring-closing reaction: The above crude product was transferred to a 100 mL round-bottomed flask, anhydrous sodium carbonate powder (31.8 g, 0.3 mol) was added to it, and the reaction was carried out under vacuum at 60 °C for 4 h, until no bubbles formed. The reaction solution was dissolved in 40 mL of fully cooled DDW and extracted with dichloromethane 3 times. The organic phase was retained and dried with anhydrous magnesium sulfate, and the filtrate was spin-dried and further dried in a vacuum oven to obtain a pale yellow powdery solid, which was M1.

(2) Homopolymerization of monomers: A solution of M1 (1.57 g, 10.0 mol) and p-toluenesulfonyl chloride (31.0 mg, 0.167 mol) in acetonitrile (5.0 mL) was added to a well-baked and nitrogen-protected polymerization flask in an anhydrous and oxygen-free environment. The mixture was heated to 80 °C and stirred for 2 h. Subsequently, DDW (0.18 mL, 10.0 mol) was added to the flask and stirred for 5 min to terminate the polymerization. The reaction solution was added dropwise into 80 mL of ether to precipitate the crude product. In order to completely remove the catalyst residue and unreacted monomers, the crude product was redissolved in acetonitrile and then added dropwise to anhydrous ether for precipitation 3 times repeatedly to obtain a white powdery homopolymer (P1).

(3) Monomer copolymerization: The copolymerization step was similar to the above homopolymerization, and the only difference was that EtOx was added as a comonomer in the feeding stage of the copolymerization reaction. Different random copolymers, P2 and P3, could be obtained with different feeding ratios (M1 and EtOx feed ratios were 1:1 and 1:9, respectively).

(4) Post-polymer modification: In order to make the polymer negatively charged, the polymers synthesized above needed to be hydrolyzed to convert the ester in the functional monomer into sodium carboxylate. The homopolymer P1 of M1 is taken as an example: A certain amount of sodium hydroxide with the equivalence ratio of it to P1 as 5:1 was completely dissolved in 3 mol/L methanol solution and cooled to room temperature. Then, P1 was added, and methanol was removed by rotary evaporation after the reaction for 6 h. The unreacted sodium hydroxide was neutralized with 1 mol/L hydrochloric acid solution, dialyzed with DDW for 48 h and then freeze-dried to obtain post-modified polymer PT1. After similar post-treatment, P2 and P3 were transformed into PT2 and PT3.

(5) Monomer and polymer characterization: The structure of monomer M1 was characterized by ^1^H NMR (Bruker, AVANCE NEO, Germany, 400 MHz, CDCl_3_), ^13^C NMR (Bruker, AVANCE NEO, Germany, 101 MHz, CDCl_3_) and mass spectrometry (Agilent, 6220, Santa Clara, CA, USA), and the structure and molecular weight of the polymer were characterized by ^1^H NMR and GPC (Waters, 515, Milford, MA, USA).

### 2.3. Preparation and Characterization of AgNPs

Catecholized chitosan was successfully synthesized according to previously reported work [27,28,29], dissolved in DDW and diluted to 1 mg/mL (according to the total volume), and it was preheated in a water bath at a constant temperature of 70 °C with continuous stirring. A pre-prepared silver nitrate solution (12 mM, based on total volume) was added and reacted for 1 h until the reaction solution was obviously discolored to obtain AgNPs.

The particle size and distribution of AgNPs were characterized using a laser particle sizer (Beckman Coulter, Delsa Nano C, Brea, CA, USA).

### 2.4. Preparation of aPOx-AgNP Multilayer Film

Preparation: Glass slides were cut into 1 cm × 2 cm rectangles evenly with a glass knife, soaked in piranha lotion (sulfuric acid: hydrogen peroxide = 3:7) for 30 min, rinsed with a large amount of ultrapure water and blown dry with nitrogen. The pretreatment steps for silicon and gold wafers were similar to those for glass slides. BPEI was formulated as a 3 mg/mL solution and adjusted to pH 9. aPOx and AgNPs were prepared in a series of solutions of different concentrations with PBS (0.01 M, pH 7.4) for use.

Assembly steps: First, the glass slide was soaked in BPEI solution for pre-assembly and taken out after 30 min, rinsed with PBS and blown dry repeatedly 3 times. Then, the glass slide was soaked in AgNP solution and taken out after 30 min, rinsed with PBS and blown dry repeatedly 3 times; then, the glass slide was soaked in aPOx solution and taken out after 30 min, rinsed with PBS and blown dry repeatedly 3 times. The above-mentioned alternate soaking assembly steps were repeated until the desired number of assembled layers was reached.

### 2.5. Layer-by-Layer Self-Assembly Behavior Tracking of aPOx-AgNP Multilayer Film

The assembly behavior of aPOx-AgNP multilayer films was tracked using a quartz crystal microbalance (QCM, Q sense, E4, Sweden). The cleaned QCM gold flakes were put into the sample cell with DDW poured in, and the frequency change of the gold flakes was observed in the wet state. After the baseline was flat, the flakes were put in BPEI solution for 30 min and poured into PBS to rinse 15 min after the frequency was stable. Then, the flakes were put in AgNP solution for 15 min and poured into PBS to rinse 15 min after the frequency was stable; then, the flakes were put in aPOx solution for 15 min and poured into PBS to rinse 15 min after the frequency was stable. The above-mentioned alternate soaking assembly steps were repeated until the desired number of assembled layers was reached.

### 2.6. Characterization of AgNPs in aPOx-AgNP Multilayer Film

The presence and content of AgNPs on the aPOx-AgNP multilayer films were detected by a UV spectrophotometer (Shimadzu, UV2550, Japan, 400 nm). The glass slide assembled with a certain number of bilayers was inserted into the slot of the UV spectrophotometer, and the blank glass slide was set as the baseline reference.

The presence, morphology and distribution of AgNPs on aPOx-AgNP multilayer films were detected by SEM (Hitachi, SU8010, Japan).

### 2.7. Characterization of aPOx-AgNP Multilayer Film

The thickness of aPOx-AgNP multilayer films was detected using an ellipsometer (J.A.Woollam, M-2000D, St, Lincoln, NE, USA). Ellipsometry is used to measure parameters such as surface film thickness and the refractive index by analyzing the change in the polarization state when light is reflected on the sample. The assembly was carried out on a silicon wafer substrate. Before the test, the multilayer film was pre-placed in a saturated humidity environment for 6 h to make the surface smooth so as to facilitate the thickness measurement. The thickness was detected in a dry state and calculated by Cauchy fitting [44], and the measurement was repeated 5 times for each experimental group.

The surface hydrophilicity of the aPOx-AgNP multilayer film was tested by a contact angle analyzer (Kruss, DSA100, Hamburg, Germany).

### 2.8. In Vitro Silver Release Test of aPOx-AgNP Multilayer Film

The experiment was divided into two parts: the control group and the experimental group. In the control group, aPOx-AgNP multilayer film glass slides assembled with a certain number of layers were immersed in PBS at a pH of 7.4. The slides were taken out and blown dry, and a UV spectrophotometer (Shimadzu, UV2550, Japan) was used to detect the content of silver on the glass slide. In the experimental group, slides with the same number of layers were immersed in PBS at a pH of 6.5 and 5.5, respectively, and the subsequent test steps were the same as those in the control group.

## 3. Results and Discussion

### 3.1. Structures of aPOx (PT1, PT2 and PT3) and M1

**Figure 4:**
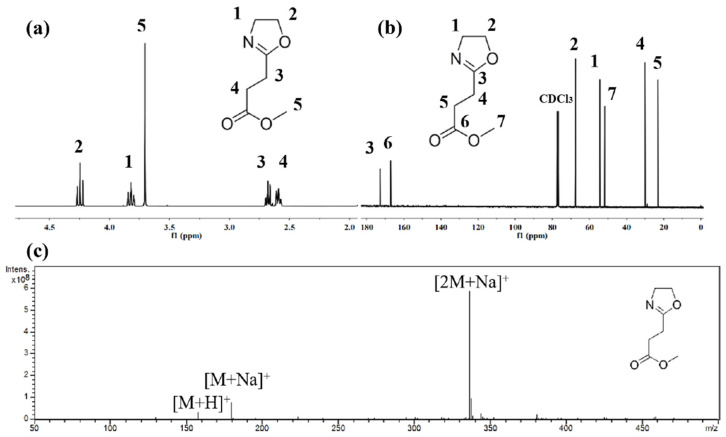
(**a**) ^1^H NMR of M1; (**b**) ^13^C NMR of M1; (**c**) mass spectrum of M1. ^1^H NMR: (400 MHz, CDCl_3_, ppm): δ = 4.24 (t, 2H), 3.82 (t, 2H), 3.70 (s, 3H), 2.68 (t, 2H), and 2.59 (t, 2H); ^13^C NMR: (101 MHz, CDCl_3_, ppm): δ = 172.74 (s), 166.99 (s), 77.41 (s), 77.09 (s), 76.78 (s), 67.47 (s), 54.36 (s), 51.80 (s), 30.02 (s), and 23.05 (s); mass spectrum (m/z): calculated relative molecular weight: 157.0; found: 157.9 [M + H]^+^, 179.9 [M + Na]^+^ and 337.0 [2M + Na]^+^.

**Figure 5:**
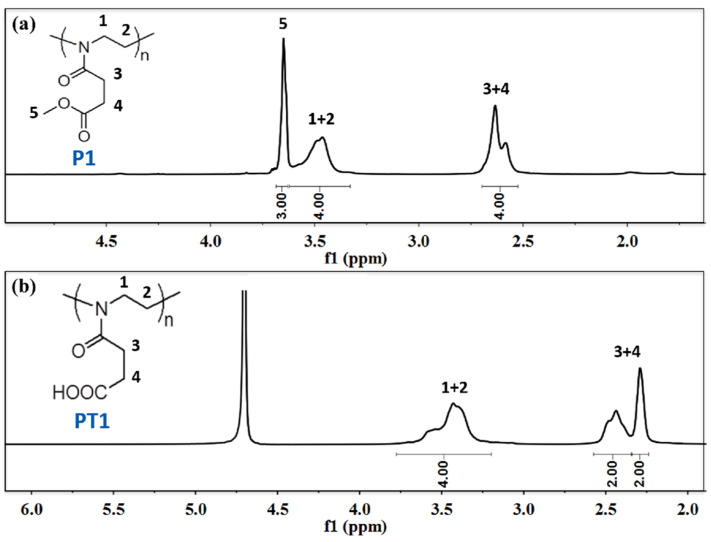
(**a**) ^1^H NMR of P1; (**b**) ^1^H NMR of PT1.

**Figure 6:**
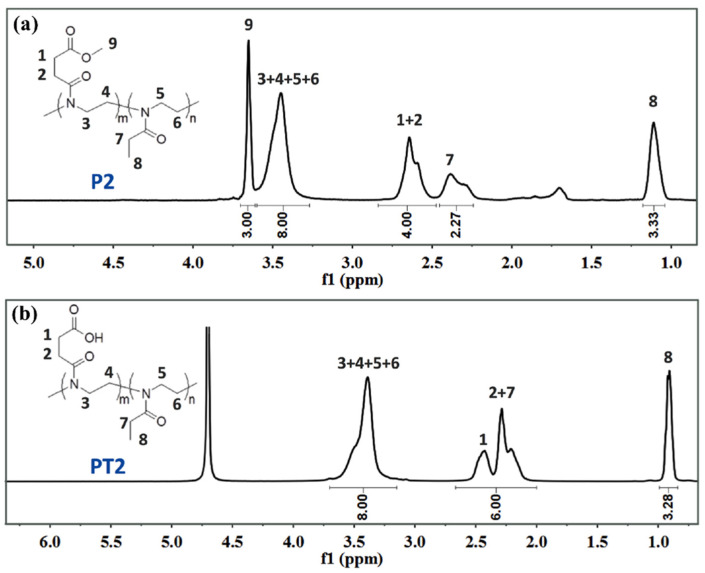
(**a**) ^1^H NMR of P2; (**b**) ^1^H NMR of PT2.

Figure 2 is a schematic diagram of the molecular formula of monomer M1, homopolymer P1, post-modified homopolymer PT1, copolymer P2 and post-modified copolymer PT2. M1 with side-chain carboxylation was obtained through chemical modification of EtOx; P1, P2 and P3 were obtained through homopolymerization or copolymerization of M1 and EtOx with adjustment of the feeding ratio, and the post-modified products PT1, PT2 and PT3 were obtained after further hydrolysis. Figure 4 is (a) ^1^H NMR of M1, (b) ^13^C NMR of M1 and (c) mass spectrum of M1, and the corresponding spectral results are consistent with the expected molecular structure. In summary, M1 was successfully synthesized with a yield of 6.9 g and 43.7%, and the related results are shown in Figure 4 and as follows:

Figure 5 and Figure 6 show the ^1^H NMR of P1 and PT1 and P2 and PT2, respectively. Compared with ^1^H NMR of monomer M1, the peaks representing two methylene groups on the oxazoline ring disappear at δ = 4.24 ppm and 3.82 ppm for the homopolymer P1, while the typical main chain methylene peak of poly 2-oxazoline appears at δ = 3.46 ppm, and the integration is correct, which means that the oxazoline monomer has successfully undergone ring-opening polymerization.

As shown in Table 1, the GPC results also show that the number average molecular weight of P1 is about 12,805, and the molecular weight distribution index is 1.197, which also assists the smooth progress of the homopolymerization reaction. In the ^1^H NMR of the post-modified product PT1, methyl peak 5 (δ = 3.65 ppm) representing the ester bond disappears, and the integration of other peaks remains unchanged, which means that the hydrolysis reaction proceeded smoothly. However, the carboxyl peak generated by hydrolysis cannot be observed due to active proton exchange, and the NMR solvent is deuterated water. Similarly, ^1^H NMR and GPC can also confirm the smooth progress of the copolymerization and post-modification reactions. In addition, as shown in Table 1, the GPC results show that the number average molecular weight of P2 is about 14,305, and the molecular weight distribution index is 1.267; the number average molecular weight of P3 is about 10,992, and the molecular weight distribution index is 1.151.

In summary, aPOx monomers (M1) and polymers (PT1, PT2 and PT3) with carboxyl groups were successfully synthesized.

### 3.2. Size and Distribution of AgNPs

**Figure 7:**
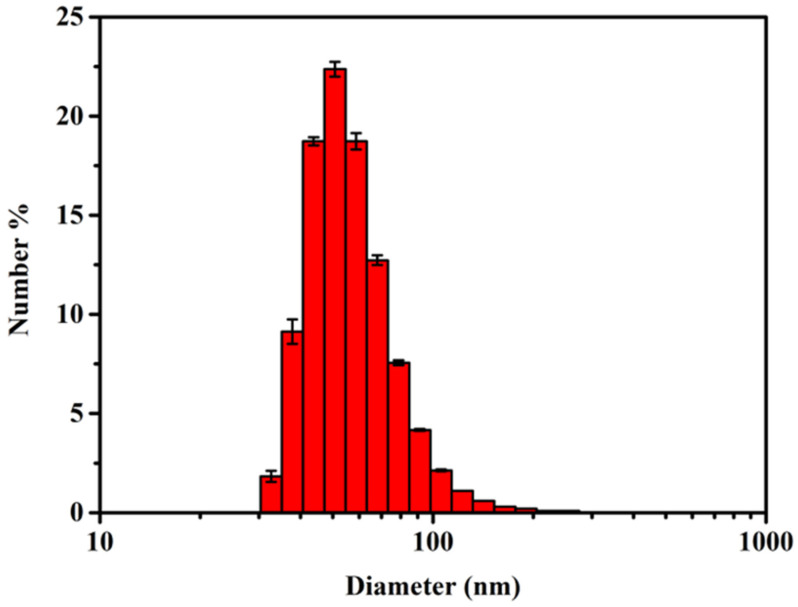
Particle size and distribution of AgNPs.

AgNPs are a kind of broad-spectrum, high-efficiency and low-toxicity bactericidal agent that has been widely studied and applied [23,24,25,26]. The AgNPs prepared by reduction and stabilization of catecholized chitosan not only have excellent in vitro cytocompatibility but also have excellent bactericidal properties against both Gram-positive bacteria (Staphylococcus aureus) and Gram-negative bacteria (Escherichia coli). Figure 7 shows the number-average particle size and distribution of AgNPs. AgNPs were successfully prepared, and the particle size mostly ranged from 25 to 175 nm. Referring to previously reported work [27,28,29], this is in line with expectations.

In conclusion, bactericidal AgNPs were successfully prepared.

### 3.3. Layer-by-Layer Self-Assembly Behavior and Parameter Adjustment of aPOx-AgNP Multilayer Film

Electrostatic layer-by-layer self-assembly is based on the electrostatic interaction between positively and negatively charged polyelectrolytes to form multilayer films through alternate deposition. The factors affecting the performance of multilayer films include the charge density, molecular weight, concentration of polyelectrolytes, and the pH value, salt concentration, etc., of the assembly solution. This study mainly explores the effect of polyelectrolyte charge density and polyelectrolyte concentration on self-assembly and the optimization of parameters.

(1) Effect of polyelectrolyte charge density on self-assembly

As the main driving force for electrostatic layer-by-layer self-assembly, the charge density of the polyelectrolyte is the key to the success of the assembly. When the charge density of the polyelectrolyte is less than the “critical charge density” [45], the assembly will not proceed. In this study, in order to be able to interact with positively charged moieties in AgNPs and electrostatically self-assemble, a carboxyl group was introduced into POx, whose main chain is electrically neutral, to obtain aPOx and make it negatively charged (become a negatively charged polyelectrolyte after ionization in aqueous solution); the charge density of AgNPs was determined and could not be changed. Therefore, only by adjusting the charge density of aPOx to make it higher than the “critical charge density” can the electrostatic self-assembly proceed. For this reason, three polyelectrolytes were prepared with charge densities, from high to low: PT1, PT2 and PT3. Keeping other assembly conditions unchanged, such as the same polyelectrolyte concentration (1 mg/mL), assembly pH of 7.4 and salt concentration of 0.01 M, PT1, PT2 and PT3 were each electrostatically self-assembled with AgNPs.

**Figure 8:**
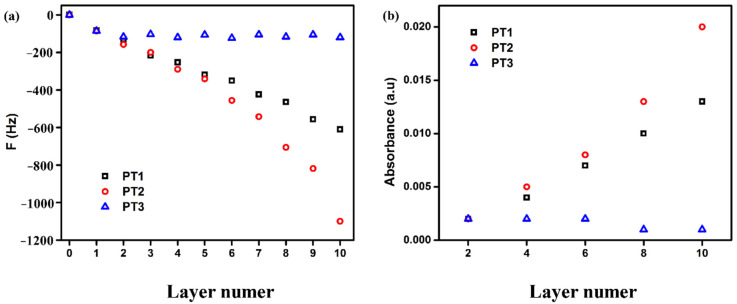
(**a**) QCM images of the self-assembly process of aPOx-AgNP multilayer films with different negative charge densities; (**b**) UV absorption images of aPOx-AgNP multilayer films with different negative charge densities.

Figure 8a is the result of the dynamic detection of the self-assembly process by QCM. The abscissa is the number of self-assembled monolayers, and the ordinate is the oscillation frequency of the wafer. The odd-numbered layers are AgNPs, and the even-numbered layers are aPOx (PT1, PT2 and PT3); the number of self-assembled monolayers is denoted by n. The change in the mass of the adsorbed substance on the wafer surface will cause a change in the oscillation frequency; that is, the change in the frequency is proportional to the change in the adsorption amount in QCM. As shown in Figure 8a, PT3, with the lowest negative charge density, has difficulty achieving continuous and effective alternate adsorption and deposition with AgNPs on the wafer, and the oscillation frequency hardly changes after only two monolayers are adsorbed; in contrast, both PT1 and PT2, with higher negative charge densities, can achieve continuous and effective alternate adsorption and deposition with AgNPs on the wafer, and the oscillation frequency continues to decrease after the adsorption of 10 monolayers. This indicates that the “critical negative charge density” in this study is between PT2 and PT3. In addition, compared with PT1, PT2, with a lower charge density, had more polyelectrolyte adsorption when self-assembled with AgNPs, and the quality of the assembled multilayer film was higher. This is because when the charge density of the polyelectrolyte is larger, the effect of charge repulsion makes the molecular chain more stretched, and the electrostatic force is stronger, forming more ion pairs (ladder conformation) with the oppositely charged polyelectrolyte [46]. Therefore, the adsorption amount is less, the formed multilayer film is thinner and more compact, and the surface is smoother. In addition, weaker electrostatic forces will reduce the number of ion pairs formed, and more polyelectrolytes have to be adsorbed to achieve surface charge reversal due to the overcompensation effect of the charge. Therefore, the lower the charge density of the polyelectrolyte (but not below the critical charge density), the greater its adsorption capacity.

AgNPs have a characteristic UV absorption peak at 400 nm, so the self-assembly process of aPOx-AgNP multilayer films can also be monitored by means of a UV spectrophotometer. As shown in Figure 8b, the UV results are consistent with the QCM results: the negative charge density of PT3 is too low, and AgNPs cannot be continuously and efficiently loaded into the multilayer film; in contrast, more AgNPs can be loaded in the multilayer film for PT2, which has the intermediate negative charge density, compared to PT1, which has the largest negative charge density. Therefore, when the charge density of the polyelectrolyte is slightly higher than the “critical charge density”, the loading effect of self-assembly is the best. In this study, PT2 and AgNPs, having shown the best assembly effect, were used in the subsequent experiments.

(2) Effect of polyelectrolyte concentration on self-assembly

**Figure 9:**
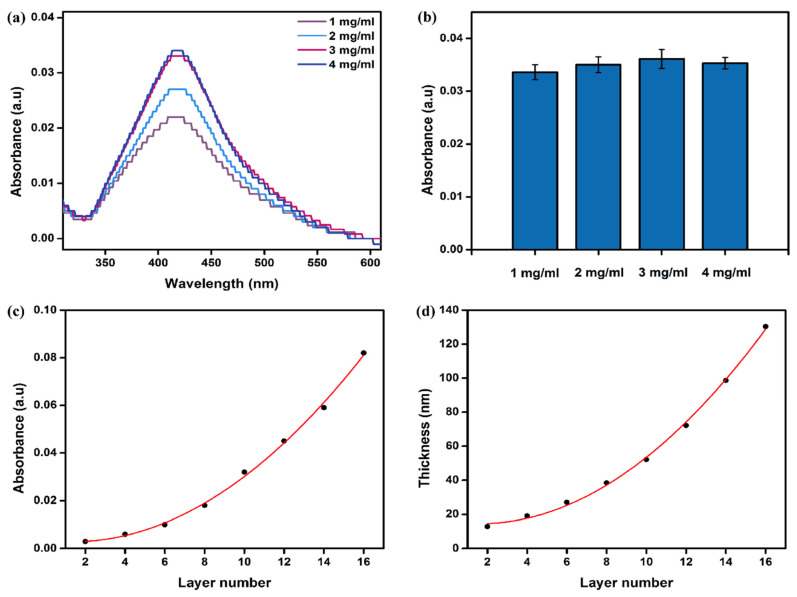
(**a**) UV absorption value when the PT2 concentration is unchanged and the AgNP concentration is changed; (**b**) UV absorption value when the PT2 concentration is changed and the AgNP concentration is unchanged; (**c**) UV absorption value of the multilayer film as the number of assembled layers increases; (**d**) thickness value of multilayer films as the number of assembled layers increases.

After determining the appropriate negative charge density, this study went on to optimize the effect of polyelectrolyte concentration on self-assembly. The aPOx-AgNP multilayer film is designed to be used for the prevention and removal of biofilms, so loading as many AgNPs as possible to improve the bactericidal ability of the multilayer film is a key parameter for measuring the bactericidal effect of the multilayer film. To this end, a series of PT2 and AgNP solutions (1, 2, 3 and 4 mg/mL) with different concentrations were prepared in this study for electrostatic layer-by-layer self-assembly (assembly time 15 min), and the assembly was measured by UV spectrophotometer for absorption values of AgNPs in aPOx-AgNP multilayer films after 10 monolayers. As shown in Figure 9a,b, when the concentration of PT2 (1 mg/mL) was unchanged, the AgNP concentration reached the peak of loading at 3 mg/mL, while when the concentration of AgNPs (3 mg/mL) was unchanged, increasing the concentration of PT2 led to a small change in the load.

In conclusion, in this study, when the concentration of PT2 is 1 mg/mL and the concentration of AgNPs is 3 mg/mL, the self-assembly effect is the best, which can maximize the loading value of AgNPs and endow the aPOx-AgNP multilayer film with the highest bactericidal power potential.

### 3.4. Properties of aPOx-AgNP Multilayer Film

After determining the optimal self-assembly parameters of aPOx-AgNP multilayer films, the properties of the multilayer films were further characterized.

The UV absorption values in Figure 9c show that the loading of AgNPs in the multilayer film increases exponentially with the increase in the number of assembled layers; the ellipsometer result in Figure 9d shows that the thickness of the multilayer film increases exponentially with the increase in the number of assembled layers. This growth mode is commonly seen in the assembly process of weak polyelectrolytes, such as natural polysaccharides [47], and is generally considered to be caused by the “interpenetration” of polyelectrolyte molecular chains in the multilayer film.

**Figure 10:**
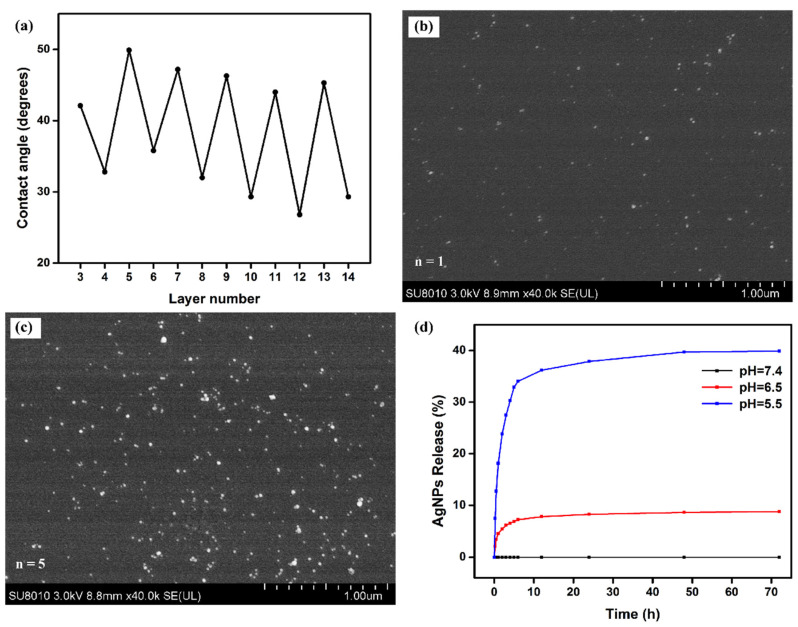
(**a**) The contact angle of the multilayer film when the number of assembled layers increases; (**b**) SEM of the multilayer film when the number of assembled layers is 1; (**c**) SEM of the multilayer film when the number of assembled layers is 5; (**d**) The multilayer film in vitro silver release test results at different pH (5.5, 6.5 and 7.4).

POx and its derivatives usually have good hydrophilicity [16,17,18,19,20], so they are often used for anti-protein adhesion. In aPOx-AgNP multilayer films, when the number of self-assembled layers is odd, the outermost layer is the AgNP layer; when the number of self-assembled layers is even, the outermost layer is the aPOx (PT2) layer. Figure 10a shows the contact angle diagram of the multilayer film when the number of assembled layers changes. The whole picture is jagged. When the number of self-assembled layers is odd, the contact angle is larger and the hydrophilicity is weaker; when it is an even number, the contact angle is smaller and the hydrophilicity is stronger, which can also indicate that the alternation of self-assembly of the aPOx-AgNP multilayer film is effectively carried out. Hydrophilicity is preferred for anti-protein adhesion.

Figure 10b,c show the SEM of the multilayer film after assembling one monolayer and the multilayer film after assembling five monolayers, respectively. The bright spherical particles on the multilayer film are AgNPs, which can also be illustrated by comparing Figure 10b,c; with the progression of self-assembly, more and more AgNPs were loaded into the multilayer film with good dispersion, and no obvious aggregation was found.

In summary, after the aPOx-AgNP multilayer film is assembled and the number of assembled layers is ensured to be even, the surface hydrophilicity of the multilayer film can be enhanced, and the anti-protein adhesion can be improved. At the same time, AgNPs are loaded to kill bacteria so as to realize bactericidal anti-adhesion potential integration of the aPOx-AgNP multilayer film.

### 3.5. pH-Responsive Silver Release of aPOx-AgNP Multilayer Film

This study further explored the pH-responsive release of silver from aPOx-AgNP multilayer films in vitro. Figure 10d shows that the release ratio of AgNPs was higher when the pH was 5.5 or 6.5, a weakly acidic condition, compared with that when the pH was 7.4. The release ratio of AgNPs was higher under the more acidic condition, which is also consistent with the weak acidity of the local microenvironment when bacterial infection occurs and is expected to be used for the pH-responsive release of silver after local bacterial infection, thereby making the aPOx-AgNP multilayer film more intelligent and efficient. The reason why the multilayer films are responsive to pH stimuli may be related to the different ionization degrees of the weak polyelectrolyte PT2 at different pH: the assembled pH of the multilayer film is 7.4, and when the assembled multilayer film is immersed in a solution with a pH less than 7.4, the ionization degree of carboxyl groups decreases at decreased pH, and the charge density of PT2 decreases, so the electrostatic interaction between PT2 and positively charged moieties in AgNPs decreases, and some AgNPs are released from the multilayer film. When the pH is 7.4, PT2 can be ionized into negative ions, and they are tightly bound to positively charged moieties in AgNPs; when the pH is 5.5 and 6.5, that is, weakly acidic, the ionization degree of PT2 decreases, and the electrostatic force with AgNPs decreases and the binding is loose, so there will be more AgNPs released from the multilayer film.

In conclusion, the bactericidal anti-adhesion potential integrated aPOx-AgNP multilayer film with the characteristic of pH-responsive silver release was successfully prepared. At the same time, self-assembly behavior tracking, parameter optimization and theoretical analysis were carried out in this study.

## 4. Conclusions

In this study, three different hydrophilic polyoxazolines with carboxyl groups (aPOx: PT1, PT2 and PT3) were successfully synthesized after monomer synthesis, polymerization and post-polymer modification, and bactericidal AgNPs were prepared by reductive stabilization of polysaccharides. The aPOx-AgNP multilayer film was prepared by electrostatic layer-by-layer self-assembly. When the charge density of the polyelectrolyte is slightly higher than the “critical charge density”, the loading effect of self-assembly is the best, and the optimal self-assembly parameters were established (PT2 1 mg/mL and AgNPs 3 mg/mL). When the number of assembled layers is ensured to be even (the outermost layer is aPOx, and the number of BPEI layers is 0), the hydrophilicity of the surface of the multilayer film can be enhanced to resist protein adhesion, and AgNPs can be loaded to kill bacteria, thereby realizing the bactericidal anti-adhesion potential integration of the aPOx-AgNP multilayer film. In addition, the aPOx-AgNP multilayer film was found to have the characteristic of intelligent and efficient pH-responsive silver release, which is expected to be used as a targeted anti-biofilm surface of implantable medical devices. Furthermore, a series of biological experiments will be arranged to gather more direct and richer evidence to explore the bactericidal activity, anti-adhesion effect and biocompatibility of the aPOx-AgNP multilayer film.

## Figures and Tables

**Table 1:** GPC of aPOx (P1, P2 and P3).

aPOx	[M1]:[EtOx]	Mn	Mw	PDI
P1	100:0	12,805	15,325	1.197
P2	50:0	14,305	18,137	1.267
P3	10:90	10,992	12,641	1.151

## Data Availability

The data presented in this study are available on request from the corresponding author.
